# World’s largest dam removal reverses coastal erosion

**DOI:** 10.1038/s41598-019-50387-7

**Published:** 2019-09-27

**Authors:** Jonathan A. Warrick, Andrew W. Stevens, Ian M. Miller, Shawn R. Harrison, Andrew C. Ritchie, Guy Gelfenbaum

**Affiliations:** 1 U.S. Geological Survey, Santa Cruz, California USA; 2 Washington Sea Grant, Port Angeles, Washington USA

**Keywords:** Sedimentology, Geomorphology

## Abstract

Coastal erosion outpaces land generation along many of the world’s deltas and a significant percentage of shorelines, and human-caused alterations to coastal sediment budgets can be important drivers of this erosion. For sediment-starved and erosion-prone coasts, large-scale enhancement of sediment supply may be an important, but poorly understood, management option. Here we provide new topographic measurements that show patterns and trends of beach accretion following the restoration of sediment supply from a massive dam removal project. River sediment was initially deposited in intertidal-to-subtidal deltaic lobes, and this sediment was reworked by ocean waves into subaerial river mouth bars over time scales of several months. These river mouth bars welded to the shoreline and then initiated waves of sediment accretion along adjacent upcoast and downcoast beaches. Although the downcoast shoreline has a high wave-angle setting, the sedimentation waves straightened the downcoast shoreline rather than forming self-organized quasi-periodic instabilities, which suggests that simple coastal evolution theory did not hold under these conditions. Combined with other mega-nourishment projects, these findings provide new understanding of littoral responses to the restoration of sediment supplies.

## Introduction

Coastal erosion and flooding hazards along the world’s shorelines pose risks to human settlements, infrastructure and natural resources^1–3^. The rates of sediment supply to coasts and the movement of coastal sediment will govern whether coastal settings accrete or erode and are, therefore, integrally related to coastal hazards^4^. Rivers are an important source of sediment to many coastal settings, and humans can increase or decrease these supplies through watershed modifications and river engineering^4–7^. Many rivers throughout the world have been dammed to provide water supplies, flood control, and hydroelectric power, which have had the unintended consequences of reducing fluxes of sediment to the coast^4,6,8^. Because river mouths and deltas are inherently dynamic landforms from the variability of river sediment supply with time as well as other hydrologic and oceanographic factors^7,9,10^, erosion of these systems can be pronounced where river sediment is intercepted by dams, such as documented for the Nile, Yangtze, Mekong, Ebro and other Mediterranean deltas^7,11–15^.

Given these coastal challenges, there is growing interest in supplementing or restoring sediment inputs to coastal systems to reduce erosion and flooding. Although dredging and beach nourishment has become routine along many developed shorelines^16^, there is increased urgency to conduct and understand innovative, large-scale projects that have the potential to reverse deficits in coastal sediment budgets and improve coastal resilience^17–19^.

**Figure 1:**
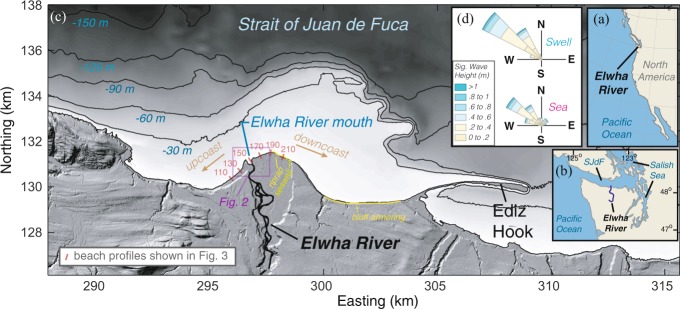
(**a**–**c**) The location of the Elwha River and its coastal setting in the Strait of Juan de Fuca (SJdF). The Elwha River littoral cell is approximately 20 km long and terminates in the downcoast direction at Ediz Hook. (**d**) Directional histograms of significant wave heights of swell and seas characterized by 4 years of wave spectra measured at two sites offshore of the river mouth. Swell and seas are separated at a threshold of 7 s wave periods (see Methods).

The removal of the Elwha and Glines Canyon Dams from the Elwha River, Washington, USA initiated an unprecedented ecosystem and sediment restoration for a river with nearly a century of reduced sediment supply^20–22^ (Fig. 1). Built in 1912 and 1927, these two hydroelectric dams were originally constructed to be 32 m and 64 m in height and with 10 million m^3^ and 50 million m^3^ storage capacities, respectively, but they did not allow fish passage for salmonids returning to spawn in the watershed. The removal of these dams, which began in 2011, can be considered the largest dam removal project to date, because it resulted in the tallest dam intentionally removed and the greatest sediment release from a dam removal project^23,24^.

Before dam removal began in 2011, approximately 30 million tonnes (Mt) of sediment was deposited in the river’s reservoirs [we use units of mass for sediment, owing to its conservation and the documented variability in sediment bulk density throughout the Elwha system^20^]. The grain size of these reservoir sediments were found to be approximately 63% coarse (sand, gravel and cobble) and 37% silt and clay by mass^25^. Sediment capture by the reservoirs resulted in downstream reductions in sediment supply that caused measurable changes in the morphology and rates of change in the fluvial and coastal landforms^26,27^. For example, reduced sediment supplies to the downstream river resulted in coarsening of much of the river bed to an armored, cobble substrate^27^. At the river delta, reduced sediment supplies resulted in shoreline erosion that averaged 0.6 m/yr during the latter 20^th^ century and increased significantly over this time^26^.

The dam deconstruction project was initiated in September 2011 and completed in August 2014. During deconstruction, the dam heights were lowered incrementally, which dropped reservoir water levels and exposed reservoir sediments to reworking by the river^20^. Within 6 months for Elwha Dam and 13 months for Glines Canyon Dam, reservoir water storage no longer existed owing to reduced dam elevations and the progradation of reworked sediment throughout the former water bodies^20^. Once sediment had progradated to the dam structure sites, the river actively spilled sediment directly into to the downstream river channel^20,22^.

In total, deconstruction of the two dams caused ~20 Mt of increased sediment supply to the river during the first five years of the project (2011–2016)^22^. This massive new supply of sediment – equivalent to two-thirds of the total sediment stored in the reservoirs – increased sediment fluxes in the Elwha River by approximately two orders of magnitude during this time^22^. Transport through the fluvial network was efficient; roughly 90% of the reservoir-eroded sediment, i.e., 18 Mt, passed though the river and was discharged to the coast^22^. Our initial mass balance showed that only about 5.4 Mt of this sediment could be accounted for in the subaerial, intertidal and subtidal regions of the river delta^21,22^. The remaining ~13 Mt of sediment was transported offshore of the submarine delta and dispersed broadly in the Strait of Juan de Fuca, largely owing to the strong tidal circulation of the region that regularly mobilizes sand and finer sediment^28–30^. In the end, the release of sediment had wide-ranging effects on turbidity, sediment flux, morphodynamics and ecosystem change within the Elwha’s riparian, estuarine and coastal systems^22,31–34^. However, the effects of this renewed supply of sediment on the morphodynamics of the broader littoral cell have not been documented.

Here we examine the relationships between the renewed river sediment supply and the morphodynamics of the Elwha River’s littoral cell by presenting new observations of topographic change. These observations are compiled to describe the rates, trends, patterns and timing of coastal changes in this littoral system. Our findings are compared with simple coastal evolution theory and other sediment nourishment projects to explore the commonalities in these results and generalizable findings.

## The Elwha Littoral System

The Elwha River (Washington, USA) delta and littoral system are in the Strait of Juan de Fuca, which connects the Pacific Ocean with the Salish Sea (Fig. 1). Wave energy arrives almost wholly from the northwest from a combination of Pacific Ocean swell and wind-generated seas from the Strait, and significant wave heights are generally less than 1 m^21,35^ (Fig. 1d). The Elwha River delta has mixed, predominately diurnal tides with a range of 2.22 m between mean lower low water (MLLW) and mean higher high water (MHHW)^35^.

The Elwha River delta protrudes seaward into the Strait of Juan de Fuca in both the subaerial and submarine topography, the latter of which provides evidence of river sediment fluxes and spit formation on the delta during lower sea levels of the Holocene^35,36^ (Fig. 1c). The littoral cell is approximately 20 km long and terminates at Ediz Hook, a modern spit associated with longshore fluxes of sediment due to the persistent oblique wave directions^35,36^ (Fig. 1). Beaches of the Elwha River delta are characterized by a steep (slope = 0.1 to 0.2) reflective foreshore backed by a berm^26^. Sediment of the foreshore and berm is mixed sand and gravel with a mean grain size of ~40 mm, and temporal variability in grain size is related to supply of sediment from the river and sediment transport in the littoral system^21,26,37^. On the downcoast section of the delta, there was a broad (~100 m), flat (slope ~ 0.02), cobble (mean grain size = 100 mm) terrace below the mean low water (MLW) elevation that formed from the erosion of the foreshore^26^, although this landform is now largely buried by new sediment. Between the river delta plain and the base of Ediz Hook, glacial-till bluffs rise landward of the beach. Their erosion is an additional source of littoral-grade (sand and gravel) sediment to the shoreline, although the rate of this sediment input has been reduced owing to ~4 km of armoring along the eastern portion of the bluffs^38^ (Fig. 1c).

As noted above, dams on the Elwha River reduced downstream fluvial sediment flux significantly^33^. In response to the reduction in river sediment flux, up to 160 m of shoreline retreat occurred along the Elwha River delta between 1939 and 2006^21,26^. Shoreline retreat resulted in the development of the cobble low tide terrace as noted above, and this naturally armored feature was formed from relic clasts exposed during the erosion of the coastal plain and also large clasts transported to the lower shoreface from kelp-mediated transport^26,35,39^.

Sediment transport along the Elwha River delta shoreline is strongly related to wave heights and breaking wave incidence angles, the latter of which varies due to the oblique approach of the waves and the curvature of the shoreline^37,40^ (Fig. 1). Littoral transport is understood to diverge at the river mouth, creating a western (or “upcoast”) subcell that has normal wave incidence angles, bi-directional sediment transport, and a cuspate shoreline^26,40^. This differs from the downcoast shoreline east of the river mouth, which has oblique breaking wave incidence angles, uni-directional downcoast sediment transport, chronic historical erosion, and geomorphic evidence of erosion from the cobble low-tide terrace and uprooted backshore vegetation^26,35,40^.

The persistent erosion of the downcoast shoreline of the river delta led to several shoreline armoring projects, including a ~600 m alongshore strip of ~0.5 m diameter boulder riprap and a 200-m long wooden seawall fronted by boulders^26^ (Fig. 1c). The 600-m stretch of riprap was continuous, but the majority of the riprap was found near the MLW elevation and detached and offshore of the beach foreshore. This section of riprap is henceforth termed “intertidal armoring.” Two short portions of the riprap connected to the shoreface, were above MHHW, and were observed to have greater effects on cross-shore and longshore fluxes of sediment. These short sections of riprap and the 200-m long wooden seawall fronted by boulders are termed “shoreface armoring” for the purposes of this study. The riprap was removed in August 2016, and the seawall was removed in April 2017, as part of a coastal restoration project.

## Coastal Response to Sediment Restoration

**Figure 2:**
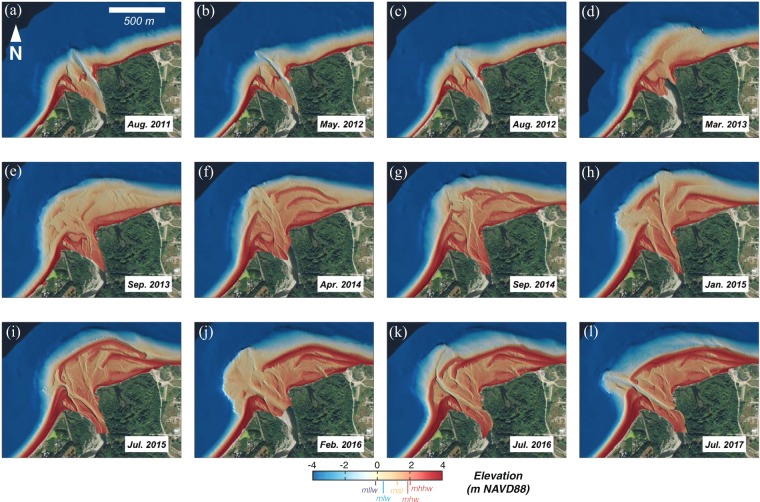
Evolution of the Elwha River delta topography and bathymetry before, during and after dam removal, 2011–2017. Elevation shown in NAVD88, and tidal datums are shown for the nearby Port Angeles water level station (see Methods). Animation of these images provided as Supplemental Materials Figure S1. Maps created with MATLAB Version: 9.2.0.556344 (https://www.mathworks.com). Base map data from the USGS National Map (Map services and data available from U.S. Geological Survey, National Geospatial Program).

To better understand the effects of the dam removals on the Elwha River littoral cell, we implemented semi-annual to annual coastal topographic and bathymetric (“topo-bathy”) surveys from 2004 to 2017 and more frequent topographic surveys at a limited number of cross-shore profiles (see Methods). Digital elevation models derived from the topo-bathy surveys reveal that the greatest changes to the littoral system occurred at the river mouth, where 100s of meters of delta progradation occurred following the dam removal project (Fig. 2; animation provided as Supplemental Figure S1). New sediment was initially deposited within the intertidal and submarine portions of the delta, and it was not until late 2013 to early 2014 – roughly two years after the initiation of dam removal – that this sediment was reworked into extensive subaerial river mouth bars (Fig. 2f).

During subsequent years, additional sediment was deposited offshore of the river mouth during high flows^22^ and was reworked by waves into river mouth bars that, in some cases, welded to the shoreline. For example, bar formation and evolution in late 2013 to early 2014 resulted in a welded bar on the upcoast side of the river mouth and two bars on the downcoast side of the river mouth (Fig. 2f). The downcoast bars welded to the shoreline between April 2014 and September 2014 (Fig. 2f,g). Additional deltaic sediment lobes were deposited from high-river flows of the 2015 winter (Fig. 2h) and the 2016 winter (Fig. 2j), and these lobes were reworked into bars during the subsequent months (Fig. 2i,k).

**Figure 3:**
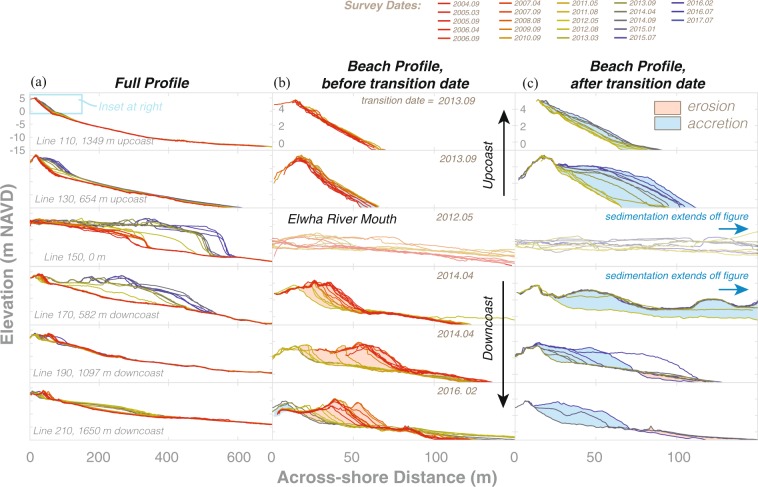
Example topographic-bathymetric profiles of the Elwha River delta shoreline. See Fig. 1c for profile locations. (**a**) Full profiles highlighting both subaerial and submarine changes with time. (**b**,**c**) Inset profiles (see top panel of (a) for inset area) highlighting the shoreface changes both (**b**) before and (**c**) after the transition dates indicating the initiation of dam removal accretion effects. Transition dates are shown in upper portions of the (b) panels. Areas of erosion and accretion are highlighted in (**b**,**c**) with shading.

The emergence and welding of new river mouth bars provided sediment directly to the adjacent beaches and broader littoral cell. The effects of these sediment sources can be observed in the cross-shore topographic profiles (Fig. 3; additional profiles shown in Supplemental Figure S2). As noted above, the largest changes occurred near the river mouth, where 5 to 10 m thick deposits extended hundreds of meters offshore of the pre-dam removal shoreline position (see Profile 150; Fig. 3a). Significant changes were also observed in beach profiles both upcoast and downcoast of the river mouth (Fig. 3b,c). Prior to the effects of dam removal, the upcoast beach profiles were relatively stable while the downcoast profiles were dominantly erosional (Fig. 3b). Following dam removal, most downcoast beach profiles in the study area transitioned to an accretionary condition, though the timing of that transition (i.e. the “transition date”) varied, and the magnitude of accretion generally decreased with distance from the river mouth (Fig. 3).

**Figure 4:**
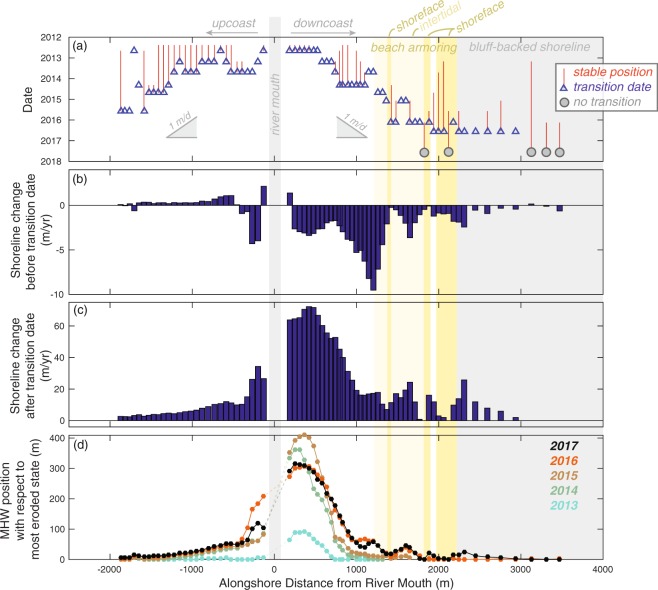
Shoreline change metrics for the Elwha River delta obtained from postions of mean high water (MHW) elevations on semi-annual cross-shore topographic profiles obtained between Sept. 2004 and July 2017. (**a**) The transition date from either stable or eroding to accretion during the post-dam removal (after Sept. 2011; see Methods). Intervals of stable beach position before the transition date are shown with red lines. (**b,c**) The rate of shoreline change (**b**) between the initial survey of 2004 and the transition date (“before transition”), and (**c**) between the transition date and July 2017 (“after transition”). (**d**) Cross-shore position of the MHW elevation contour during summer (July-Sept) topographic surveys with respect to the most eroded (i.e., landward) state of the profile.

We investigated the timing of the transition from either stable or erosional to accretional for the study area by evaluating time series of the cross-shore positions of the mean high water (MHW) elevation, the standard datum for shoreline position assessments in the U.S. and a recognized local proxy for the shoreline^26^ (see Methods). Transition dates ranged from 2012 (~1 year after the initiation of dam removal) near the river mouth to 2016 (5 years after the initiation of dam removal), at sites 2 to 3 km from the river mouth (Fig. 4a). These measurements suggest that the propagation of this accretional response was approximately 1 m/d in both directions from the river mouth (Fig. 4a). As of 2017, transitions were not observed in some of the downcoast profiles, suggesting that these profiles had no significant accretion during these post-dam removal measurements. Two of these profiles were located in sections of the beach with shoreface armoring and three were located on bluff-backed beaches over 3000 m from the river mouth (Fig. 4a).

Rates of shoreline change increased markedly after the transition dates. The average rate of change of shoreline position during the pre-transition period (i.e., between our earliest survey in 2004 and the date of transition) ranged from slightly accretional for the majority of the upcoast beaches (generally ~0.3 m/yr) to strongly erosional for the downcoast beaches (up to 2–9 m/yr, although less for reaches with shoreface armoring or coastal bluffs; Fig. 4b). Rates of erosion of the downcoast beaches were much greater than the 0.6 m/yr average rate reported for the latter 20^th^ century, which is consistent with the hypotheses of acceleration in erosion during the 21^st^ century^26^. After the transition dates, shoreline change rates were accretional throughout the study area, exceeding tens of meters per year for some profiles in the study area (Fig. 4c). For the upcoast beach profiles, rates of accretion increased by over an order of magnitude (from 0.2-to-1.0 m/yr to 3-to-20 m/yr; Fig. 4).

A time series of relative shoreline positions shows shoreline progradation that exceeded 100 s of meters near the river mouth and was 10 s of meters for the distal beaches (Fig. 4d). Near the river mouth the maximum progradation was observed in 2015, after which the shoreline retreated (Fig. 4d). This 2015-to-2017 retreat coincided with the onset of accretion at adjacent beaches, for example, at 800–2000 m downcoast from the river mouth (Fig. 4d). The spatial patterns of accretion were more irregular on the downcoast beaches than the upcoast beaches, however, as shown by spatial and temporal cycles of the relative shoreline position (Fig. 4d). The total accretion along the upcoast beaches generally decreased monotonically with distance from the river mouth, whereas undulations in accretion were observed in the downcoast relative shoreline positions (Fig. 4d). We investigate these phenomena in the following section with additional beach profiling at higher temporal frequencies.

## Observations of Coastal Sedimentation Waves

**Figure 5:**
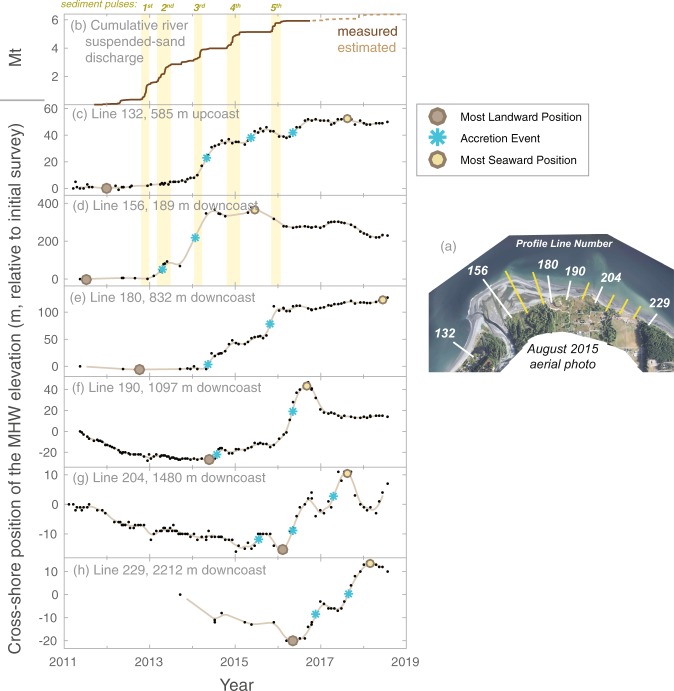
Time series of shoreline positions derived from regularly surveyed topographic profiles along the Elwha River delta. (**a**) Location of the twelve profiles sampled, data from six of which (white lines) are plotted in (**c**–**h**). The remaining profiles (yellow lines) are shown in Supplemental Materials. (**b**) The Elwha River suspended-sand discharge from Ritchie *et al*. (2018) with discharge events highlighted with vertical bars. (**c**–**h**) Small symbols show the cross-shore postions of mean high water (MHW) elevations from topographic profiles; lines provide quarterly moving averages. Accretionary events and cross-shore minimum and maximum are shown with symbols (see Methods). Note that each vertical scale in (**c**–**h**) is unique. Inset map created with ArcMap version 10.5 (http://desktop.arcgis.com/en/arcmap/) using structure-from-motion products created with Agisoft PhotoScan 1.1.6 through 1.2.6 (http://www.agisoft.com).

For twelve sites around the Elwha River delta, topographic profiles were measured with up to biweekly frequencies, from which time series of MHW shoreline positions were determined (see Methods). Time series of shoreline positions for half of these profiles are shown in Fig. 5 (all data are shown in Supplemental Figure S3). Additionally, the timing of the most landward and seaward positions of the shoreline and the timing of shoreline “accretion events” as identified by the maxima in the rates of shoreline accretion (see Methods) were identified from shoreline position time series of each profile (Fig. 5).

Time series of shoreline position provide evidence that the Elwha River beaches did not accrete in a steady manner, but rather accreted in punctuated patterns with several discernable episodes over intervals of weeks to months (Fig. 5). Only one upcoast profile was sampled with these higher temporal frequencies, and it exhibited three distinct accretion events, each occurring between 2 and 6 months after pulses in the river sediment flux (Fig. 5b,c; yellow bars). The downcoast profiles exhibited intervals of both erosion and accretion that were not uniform in magnitude or synchronous in time across the sites. In fact, the first accretion event at the profile adjacent to the river mouth (Line 156) occurred 2 to 3 years before the first accretion event identified at more distal downcoast profiles (Lines 204 and 229; Fig. 5).

**Figure 6:**
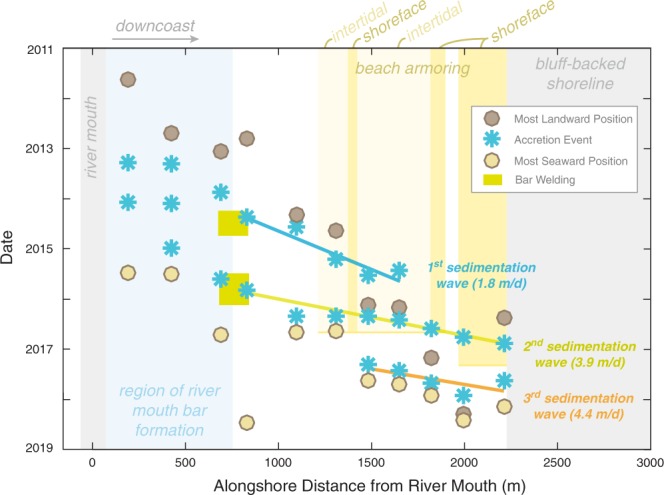
Shoreline change metrics for the Elwha River delta obtained from postions of mean high water (MHW) elevations on biweekly across-shore topographic profiles obtained between 2011 and 2018. Rates of change computed by linear regression through all data points downcoast of bar welding location (shown with green bars). Bar welding timing and location obtained from topographic data shown in Fig. 2. Yellow vertical bars show the different sections of beach armoring and terminate at the date of removal.

A compilation of the timing of accretion events from the frequently sampled beach profiles, as well as the timing of the most landward and seaward shoreline positions, shows fairly systematic patterns in their downcoast propagation (Fig. 6). Additionally, two downcoast accretion events align with the timing and location of two major river mouth bar-welding events in mid-2014 and in late-2015 (Fig. 6). This provides support for a hypothesis that bar welding, which was driven by cross-shore and longshore sediment transport phenomena, initiated downcoast waves of accretion. The first of these waves becomes indistinguishable after ~2 years and ~1800 m distance from the river mouth (Fig. 6), although this may be a function of limited sampling in this region during this time (*cf*. Supplemental Figure S3). Additionally, the dissipation of this wave occurs within the armored section of coast, which may have played a role in attenuating this sedimentation wave. The magnitude of accretion also decreased with distance from the river (it was greater than 10 s of meters at Line 180 and less than 4 m at Line 204; Figs 5, 6). The second wave was generally greater in magnitude (~20 m of shoreline accretion across the entire study area) and transpired over an interval of ~1.5 years (Fig. 6; *cf*. Figure S3 in Supplemental Materials).

Because of the downcoast propagating behavior of these accretion events, we describe them as ‘sedimentation waves’ (Fig. 6). This terminology was chosen over the commonly used ‘sand waves,’ which generally describes migrating and undulating shoreline morphology^41–43^, because we observed overall straightening of the shoreline as shown below. Downcoast of the bar welding locations, the first two sedimentation waves propagated at average speeds of 1.8 and 3.9 m/d, respectively. There was also evidence of a third sedimentation wave that formed roughly 1500 m from the river mouth in early 2017 (Fig. 6). This third sedimentation wave formed within the region of shoreline armoring – not at a welded river mouth bar – and its formation followed the removal of the armoring in late 2016 and early 2017 (Fig. 6). One hypothesis is that armor removal may have liberated littoral sediment owing to groin-like characteristics of the shoreface portions of these structures, although a direct confirmation of this hypothesis is not possible with our data.

**Figure 7:**
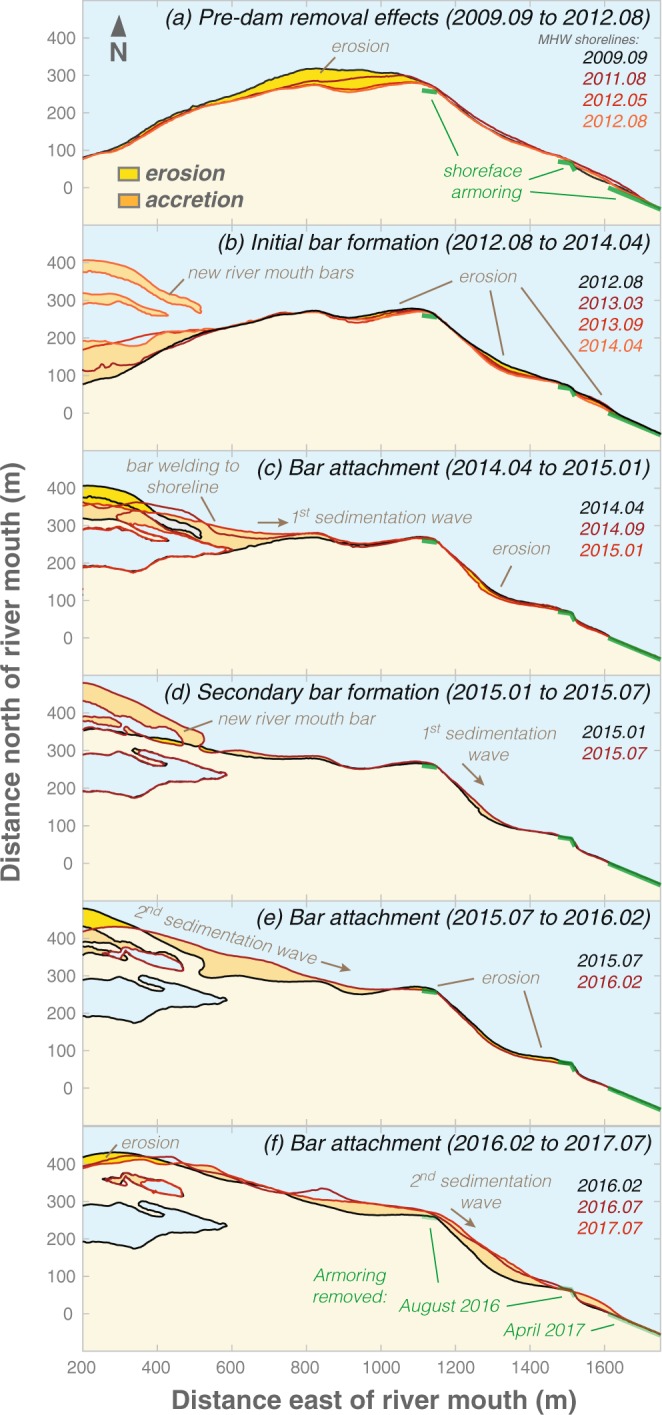
Shorelines of the downcoast Elwha River delta before, during and after dam removal showing river bar welding and subsequent sediment waves. Shorelines derived from the MHW contours extracted from DEMs generated by the topo-bathy surveys.

The spatial and temporal evolution of the shoreline from our topo-bathy surveys provides additional insights into the initiating mechanisms and propagation of these downcoast sedimentation waves (Fig. 7). When the first sub-aerial river mouth bar formed in early 2014, the downcoast shoreline was chronically eroding except for locations with shoreface armoring (Fig. 7a,b). Welding of this initial bar to the shoreline produced the first sedimentation wave that could be traced downcoast to the westward end of the shoreface armoring (Fig. 7c) and then to an area within the shoreface armoring (Fig. 7d). After this first sedimentation wave passed, much of the downcoast shoreline returned to an erosional state, except where shoreface armoring existed (Fig. 7e). Formation and welding of the subsequent river mouth bar resulted in the larger, second sedimentation wave that propagated down the shoreline (Fig. 7d–f).

Although the upcoast portion of the littoral cell also exhibited three distinct accretion events (Fig. 5c), this region of shoreline exhibited relatively simple monotonic accretion that decreased with distance from the mouth (see mapped shorelines in Supplemental Figure S4). Unfortunately, our limited observations of the upcoast shoreline profiles inhibited us from evaluating whether accretion in this portion of the system propagated in sedimentation waves.

Overall, the processes of delta expansion, river mouth bar formation and welding, and accretion within the littoral cell increased the volume of sediment in these systems significantly. We assessed the magnitude of this change by calculating survey-to-survey topographic differences, and used elevation bounds to isolate the active beach portion of the study area (see Methods). These computations suggest that the total increase in coastal and marine sediment was approximately 3.6 million m^3^ (or ~5.4 Mt using estimates of sediment bulk density for this beach^20^), but that only 20-25% of this sediment (~800,000 m^3^ or ~1 Mt) was in the intertidal and subaerial portions study area (see Supplemental Figure S5). Thus, the broad expansion of the beaches of the littoral cell was caused by only a small fraction of the total 20 Mt of sediment released by the dam removal.

## Discussion

Deltas throughout the world are increasingly vulnerable to erosion and flooding from human activities that have reduced sediment inputs, compacted existing sediments, and increased sea levels^6,15,44^. Restoring natural sources of sediment to these coastal systems has the potential to build landmasses and reduce coastal hazards^19,45^. Here we examine the new understanding of coastal morphodynamics gained from the Elwha River delta and assess the implications of these findings to other coastal systems worldwide.

### Morphodynamics of the restored elwha river delta

The Elwha River coastal system responded rapidly to the reintroduction of millions of tonnes of sediment formerly stored within its reservoirs. The coastal morphodynamic response was initiated with submarine lobes of coarse sediment deposited at the river mouth, the upper portions of which were reworked by ocean swell and wind waves during the pending months to years into subaerial river mouth bars (Figs 2, 3). Sediment input was commonly greatest during high river discharge of the winter^22^ (November to February; Fig. 5b), which resulted in bar formation by the subsequent summer (June to July; Fig. 2).

The welding of these subaerial river mouth bars to the shoreline provided a direct link between the fluvial sediment inputs and the delta’s littoral cell. Although the upcoast and downcoast shorelines both exhibited pulsed accretion events (Fig. 5), the morphodynamic response of these beaches was different. Because of the near zero breaking wave incident angles upcoast of the river mouth^26,40^, the upcoast shoreline exhibited diffusive behavior in response to the new sediment, and the magnitude of shoreline change was inversely related to the distance from the river mouth (Fig. 4; Fig. S6 in Supplemental Materials). This response is similar to the diffusional, low-wave-angle delta morphodynamics suggested by coastal evolution theory and sediment transport models^46–48^.

In contrast, the oblique wave approach to the downcoast shoreline (Fig. 1) results in breaking wave incident angles directed toward the east, which strongly drives sediment transport in the downcoast direction^21,37,40^. In fact, the deep-water incident wave directions for much of the downcoast shoreline are greater than the 45**°** threshold defining “high-angle” coasts, which suggests that self-organized, quasi-periodic, shoreline instabilities such as spits and sand waves should be expected^42,46,48^ (see Supplemental Figure S6).

Although we documented the formation and translation of several sedimentation waves along the downcoast shoreline (Figs 6, 7), these features were fundamentally different from the shoreline instabilities predicted by general coastal evolution theory^42,46,48^. Sedimentation waves at the Elwha generally became less pronounced with distance and time, and they tended to straighten the shoreline (Fig. 7). This contrasts with the general theory that instabilities become more pronounced after initiation thereby elongating the shoreline with time^42,47^. Furthermore, the strongly oblique and unidirectional nature of the incident waves would predict spits that propagate downcoast (i.e., “flying spit” shoreline forms^42^), which is fundamentally different from the straightening shoreline we observed. For example, for the downcoast portion of the Elwha River delta littoral cell, the fraction of waves that are high-angle is ~1 and the fraction of waves approaching from a single high-angle quadrant is ~1 (*cf*. Figure 1 and Supplemental Fig. 6). General theory suggests that these conditions should result in flying spits for the downcoast shoreline^42^, a theoretical prediction confirmed with the use of a simple shoreline evolution model scaled for the Elwha River delta (see Supplemental Figure S7). The discrepancy between our observations and general theory is not related to breaking wave incident angles, which are regularly observed to be high-angle^21,26,40^. Rather, other factors may lead to this discrepancy. For example, the complex bathymetry of the Elwha submarine delta and the broader Strait of Juan de Fuca causes wave refraction that is suggested to result in increases in wave heights with distance downcoast of the Elwha River mouth^21,35^, and these patterns are fundamentally different from the decreasing breaking wave height patterns predicted from the wave refraction models used in simple coastal evolution models^42,47,48^ (cf. Figure Supplement Fig. 6d). Additionally, the strong tidal currents of the Elwha River delta setting, which are responsible for export of the majority of sediment off of the submarine delta^21,28–30^, may inhibit sand wave and spit formation by preventing the submarine formation of these features.

Thus, the littoral response to massive increases in sediment supply varied spatially and in time and was generally inconsistent with simple theories of beach and delta morphodynamics for high-angle wave environments. This suggests that assessments of the long-term (decadal to millennia) trajectory of this river delta – which will be important in assessing future coastal evolution and flooding – will need to use different predictive approaches to understand the pending evolution of this sediment and the shoreline. It is likely that the cumulative effects of multiple sedimentation waves and their spatial and time-dependent transformation throughout the littoral cell will determine the position, shape, potential hazards, and habitat characteristics for the downcoast beaches in the years and decades to come, and predictive capabilities will need to incorporate these sediment transport phenomena to adequately characterize coastal evolution.

### Implications for restoring deltas and shorelines

At the same time that coasts throughout the world are being challenged by erosion and flooding hazards, the storage of river sediment behind human-made dams continues to increase^49^. On global scales, an estimated 100 billion tonnes of sediment are stored behind dams, and this increases by over a billion tonnes per year as new dams are built and existing reservoirs continually fill with sediment^4,50^. All dams have operational lifespans and will need reengineering, desilting, replacement, or eventual removal. Thus, there are increasing opportunities to realize the benefits of restoring sediment supplies to river and coastal systems^23,24,51^.

Large-scale human interventions to restore sediment to coastal systems are becoming more attractive with time as large pilot projects such as the dam removal presented herein and the mega-nourishment ‘Sand Engine’ on the Netherland’s shoreline are implemented and studied^17,18^. With respect to river deltas, many of these systems have experienced centuries or millennia of human impacts from activities such as human occupation and development of the watershed^5,7^. Additionally, recent measurements show expansion of some deltas in regions experiencing increased watershed sediment production owing to climate change, land use, or other perturbations^4,6,52^. As such, humans – whether directly or indirectly – have long been agents of change in the morphodynamics of the deltas of the world^7^. Hence, continued human actions through sediment restoration or enhancement will likely be necessary to reduce future coastal hazards of erosion and flooding, especially as coastal populations increase and sea levels rise.

For future projects, it will be important to understand the implications of restoring sediment to coastal systems. With respect to the long-term viability of land generated from accretion, the decadal-scale effects of sediment restoration projects will be important to document and understand. Unfortunately, insufficient time has transpired for the Elwha River or the Sand Engine projects to document these longer time scales. The initial (2–5 yr) results from these projects do show significant similarities, however, including rapid initial changes as ocean waves reshape the new sediment, downcoast-dominated transport, and decreasing shoreline response with distance from the project^17,18^. Combined, these findings provide important clues for how future sediment restoration events may influence coastal morphology. These findings are also generally consistent with smaller beach nourishment projects in open coastal settings, although for many of these settings the annual cross-shore exchange of sand can rival total nourishment volumes making the identification and persistence of the effects of these projects more difficult to detect^53,54^. Although our simple application of coastal evolution models were only partially successful at describing shoreline change of the Elwha River delta, the use of process-based approaches, such as used for the Sand Engine^18^, have shown excellent potential to predict sediment transport and geomorphic change. Future sediment restoration projects may gain important predictive capabilities from taking similar process-based approaches.

Although our findings suggest that sediment restoration actions may have significant benefits to reducing coastal erosion, these benefits must be weighed against economic, cultural and ecological implications and the financial costs of these projects. In this light, detailed observations and models of the riparian, estuarine and marine ecosystems of the Elwha^31,32,55^ should provide useful tools and concepts for assessing other systems. Additionally, as more dam removal and sediment restoration projects are completed, syntheses are being developed outlining controlling factors, commonalities and differences of outcomes^23,24^.

In conclusion, the results presented here suggest that the effects of restoring sediment to coastal systems can be rapid and measurable, and that they can significantly change the projection of a river delta shoreline and its adjacent littoral cell. However, our measurements suggest that the morphodynamic response of the Elwha River littoral cell was not simple and was not entirely consistent with simple coastal evolution theory. Thus, these measurements provide essential understanding of the scale, timing and patterns of the geomorphic effects of one of the largest sediment restoration events in world history.

## Methods

Wave climatology for the Elwha River delta was characterized from acoustic Doppler current profilers (ADCPs) deployed offshore of the river mouth between 2010 and 2014^56^. Wave energy spectra were converted into wave statistics (significant wave heights, dominant period, dominant direction) for both swell and seas, which were defined by separating the spectra at 7 seconds periods.

Topographic and bathymetric surveys (“topo-bathy”) were conducted at semi-annual to annual intervals during coordinated land and ocean campaigns. Nearshore bathymetry surveys were performed using personal watercraft, small boats, and a kayak, each equipped with single-beam echosounders and survey-grade global navigation satellite system (GNSS) receivers. Topography data were collected by walking along survey lines with GNSS receivers mounted on backpacks. Positions of the survey platforms were differentially corrected to a GNSS base station placed on a nearby benchmark with known horizontal and vertical coordinates. To ensure data overlap, bathymetry measurements were taken during higher tidal stages, and topography measurements were taken at lower tidal stages.

Nearshore bathymetry and topography data were primarily collected along a series of shore-perpendicular profiles spaced at 25–50 m intervals along the coastline and extending from landward of the primary beach berm crest to approximately 10–16 m water depth. However, early surveys (2004–2006) only sampled every 2^nd^ to 4^th^ profile (50–100 m profile spacing). Starting in 2009, additional topographic and bathymetric sampling occurred between profiles, including along topographic features such as breaks in slope. With the higher data density beginning in 2009, linear interpolation was used to construct seamless digital elevation models (DEMs) from all available survey data. All survey data and DEMs are available in a USGS publications^57,58^.

In addition to these comprehensive surveys, more frequent topographic surveys were conducted on twelve cross-shore profiles (see Supplemental Figure S3). These more frequent surveys utilized a GNSS receiver mounted on a survey rod. Survey frequency varied considerably, however, owing to weather, equipment issues, accessibility to the sites, and other factors. These profile data are available from a separate data release^59^.

Shoreline positions were determined for each profile and the mapped DEM products by determining the location of mean high water (MHW) elevation in the topography. The MHW elevation typically bisects the shoreface of the Elwha River delta beaches^26^. The NOAA Tidal Station at Port Angeles, Washington (Station Number 9444090) was used to determine all tidal datum, including the MHW elevation of 1.86 m NAVD88.

Time series of the MHW shoreline positions were used to determine several metrics of change from the profiles. For the semi-annual to annual surveys, we determined the post-dam removal date of transition from stable or erosional to accretion by identifying the most landward (most eroded) shoreline position in the MHW position time series. Only the post-dam removal record was used for this analysis (2011 to 2017). Additionally, we applied a horizontal shoreline position uncertainty of 2 m, which was computed from the combined vertical and horizontal uncertainty of the GNSS survey (~10 cm), the average slope of the beach (~0.1), and the assumed horizontal position error from the walked survey of the profile (~1 m). The transition date was defined to be the final (or most recent) date within this 2 m uncertainty of the most landward position of the shoreline. If there were multiple shoreline measurements within the uncertainty of the most landward position, the shoreline was classified as ‘stable’ for these dates, and this condition was shown with vertical lines in Fig. 4a. The average rates of change before and after this transition date were measured from the complete time series of MHW positions (2004 to 2017) using the slope of linear regressions. Additionally, the relative shoreline positions shown in Fig. 4d were defined by subtracting the most landward position of the shoreline from the time series of the MHW positions.

For the twelve profiles surveyed more frequently, we developed additional metrics of change using cross-shore MHW positions. For these profile data, we used a 3-month low-pass filter to develop continuous weekly records of shoreline position with a focus on seasonal-scale changes, and we developed several metrics from these low-pass records (raw and low-pass records for all profiles are presented in Supplemental Figure S3). The dates of the most eroded and accreted state of each profile were taken from the most landward and seaward position, respectively, of the time series. “Accretion events” were defined to be continuous intervals of time with monotonic, seaward movement in the low-pass shoreline position. Accretion events were limited to only those that had total shoreline change of at least 20% of the total difference between most eroded and accreted shoreline position, which limited the number of events to 2 or 3 per profile. The timing of each accretion event was defined by the point of maximum rate of seaward growth in the low-pass time series.

## Supplementary information

All Supp. Info.

Supplemental Figure 1

## Data Availability

All data are available at published and accessible U.S. Geological Survey and Pangea sources^57–59^.
