# Supplementary material for: World’s largest dam removal reverses coastal erosion
Source: Sci Rep. 2019 Sep 27;9:13968. doi: 10.1038/s41598-019-50387-7 (PMC6764949; doi:10.1038/s41598-019-50387-7)
Supplement: Supplementary file 1 — All Supp. Info. [file 41598_2019_50387_MOESM1_ESM.pdf]

Supplementary Information

World's largest dam removal reverses coastal erosion

Jonathan A. Warrick[1#], Andrew W. Stevens[1], Ian M. Miller[2], Shawn R. Harrison[1], Andrew C. Ritchie[1], Guy Gelfenbaum[1]

[1]*U.S. Geological Survey, Santa Cruz, California, USA*

[2]*Washington Sea Grant, Port Angeles, Washington, USA*

Manuscript: SREP-19-20916A

Submitted to: *Scientific Reports*

[#]*corresponding author:*

Jonathan A. Warrick

Pacific Coastal and Marine Science Center

U.S. Geological Survey

2885 Mission St.

Santa Cruz, CA 95060, USA

Email: jwarrick@usgs.gov

Introduction

Several supplemental figures are provided to assist with a more comprehensive presentation of the data and findings. Supplemental Figures S1 to S5 provide additional details for data and figures already presented in the main paper as summarized below:

Supplemental Figure S1 provides an animated GIF of the Elwha River delta topo-bathy DEMs presented in Fig. 2. Maps created with MATLAB Version: 9.2.0.556344 (https://www.mathworks.com). Base map data from the USGS National Map (Map services and data available from U.S. Geological Survey, National Geospatial Program). Animation made with Camtasia 2018 (https://www.techsmith.com/video-editor.html).

Supplemental Figure S2 is identical in format to Fig. 3, but it provides twice as many topo-bathy profiles.

Supplemental Figure S3 provides the complete set of MHW shoreline positions for the twelve frequently surveyed profiles, and it uses the same format as Fig. 5.

Supplemental Figure S4 provides the mapped MHW shorelines for the upcoast portion of the study area to contrast with the downcoast shorelines that are presented in Fig. 6.

Supplemental Figure S5 is a time series of cumulative volume change for the Elwha River delta study area. The littoral portion is defined by volume change above MLLW elevation.

The remaining two figures, Supplemental Figures S6 and S7, provide information about wave transformation and sediment transport patterns of the Elwha River delta as predicted by the shoreline evolution theory of Ashton and Murray[1]. These results are presented for comparative purposes with our observations of coastal change at the study site:

Supplemental Figure S6 provides a number of parameters for the Elwha River delta from the formulations of Ashton and Murray[1] including: deep-water and breaking wave incident angles, breaking wave height, and relative alongshore sediment flux.

Supplemental Figure S7 provides an example output of a shoreline evolution model developed by Roelvink et al.[2] based on the theory and formulations of Ashton and Murray[1].

Further information about these figures is provided below.

Supplemental Figure S6 – Wave and Transport Parameters

General wave and sediment transport parameters were generated from shorelines derived from the topo-bathy surveys of the Elwha River delta and wave observations from immediately offshore of the river mouth. Here we show results from the July 2015 shoreline and a mean offshore wave direction of 310°. The shoreline was connected across the river mouth and smoothed with a 10-meter low-pass filter to produce a continuous and regular feature (Fig. S6a).

The incident direction of the deep-water waves (θ_0) were computed from projecting wave direction (310°) upon the local shoreline normal (Fig. S6b). For

these calculations, positive values were defined to be upcoast, and negative values were defined to be downcoast. Additionally, we added the theoretical shoreline morphodynamic zones based on incident angles reported by Ashton and Murray[1], which include diffusive, high-angle, and negligible transport zones (Fig. S6b). The alongshore position of these zones are highlighted with shading in Fig. S6. We also include 10° of variance in the wave directions as shown by shading about these results.

Breaking wave parameters (Fig. S6c,d) were computed using the formulations of Ashton and Murray[1] and additional formulations of Larson et al[3]. It is noted that these formulations assume simple parallel bathymetry to the shoreline. The incident direction of the breaking waves (θ_b) and the breaking wave heights (h_b) were calculated by:

$$\theta_b = \arcsin\left(\sqrt{2\pi}\ \sin\theta_o\ \sqrt{\frac{h_b}{L_0}}\right) \qquad [1]$$

$$h_b = L_0\left[\left(\frac{H_o}{L_0}\right)\frac{\cos\theta_0}{\gamma_b^2 2\sqrt{2\pi}}\right]^{2/5} \qquad [2]$$

where L_0 is the deep-water wavelength solved by linear wave theory, H_0 is the deep-water wave height, and γ_b is the wave breaker depth ratio, which was assumed to be 0.83. For our calculations, H_0 was 0.4 m and the wave period (T) was 10 s.

An estimate of relative alongshore sediment transport was made with these parameters using the CERC-style breaking wave formulations of Ashton and Murray[1]:

$$Q_s = \mathrm{K}_1\ H_b^{5/2}\cos\theta_b\sin\theta_b \qquad [3]$$

where K_1 is an empirical constant. We computed the relative sediment transport ($Q_s/Q_{s\,max}$) along the study area by setting K_1 to an arbitrary value and dividing all computed values of Q_s by the maximum computed value of the study area ($Q_{s\,max}$; Fig. S6e).

Supplemental Figure S7 – Coastal Evolution Model

We utilized the free-form coastline evolution model, *ShorelineS*[2], to explore the morphodynamic patterns predicted by the principles derived from general coastline theory and the Coastal Evolution Model[1]. The model allows for shoreline behaviors including the nonlinear undulations and spits resulting from in high-angle wave scenarios[1,2]. Matlab code for the model is available with documentation at www.shorelines.nl and https://github.com/danoroelvink/shorelines.

We explored the use of this model to describe the Elwha River delta morphodynamics by using our mapped shorelines and approximations of the wave fields for durations of 0.5 to 2 years of model time. We ran dozens of model scenarios, and they resulted in similar patterns of morphodynamic changes to the shoreline. These patterns included diffusive spreading of sediment upcoast of the river mouth and the formation of high-angle shoreline instabilities downcoast of the shoreline (Fig. S7). These patterns are is consistent with the general parameters and theory presented in Fig. S6.

Models were initiated with the July 2015 shoreline, and a wide range of parameter values were used, including run lengths of 6 months to 2 years, wave

heights from 0.2 to 1 m, mean wave directions from 295° to 325°, wave spreading angles of 0° to 60°, initial model domain spatial spacing of 5 to 50 m, active beach profiles of 2 to 10 m in elevation, and minimum spit widths of 10 to 100 m. All runs utilized a standard CERC formulation for sediment transport rates. The model runs produced consistent patterns of diffusive spreading upcoast of the river mouth and sand wave to spit formation downcoast. However, we did find that the wavelength of the sand waves and spits generated in the model runs increased with increasing initial model spacing.

An example output from the model is shown in Fig. S7, for which the evolution of the coast was allowed for 6 months with constant 0.5 m wave height. Wave direction was 310° with a maximum spread of ±30°. This model was initiated with model spacing of 10 m, an active beach profile height of 3 m, and a minimum spit width of 20 m. The final shoreline reveals several flying spits downcoast of the river mouth with wavelengths of approximately 400 m. We note that this wavelength is smaller than the typical sizes of 1-10 km reported by Medellin et al.[4]. However, it is consistent with scaling developed by Falque and Calvete[5] that suggests that the geomorphic wavelength should be 40-150 times the surfzone width. Applied to the Elwha River delta, which has a surzone width of only 3-5 meters owing to its steep, reflective beach and small waves[6], the flying spit wavelengths are predicted to be only 150-750 m, which is consistent the with results shown in Fig. S7.

References Cited

1. Ashton, A. D. & Murray, A. B. High-angle wave instability and emergent shoreline shapes: 1. Modeling of sand waves, flying spits, and capes. *J. Geophys. Res.* **111**, (2006).
2. Roelvink, D., Huisman, B. A. & Elghandour, A. Efficient modeling of complex coastal evolution at monthly to century time scales. *Proc. Sixth Int. Conf. Estuaries Coasts ICEC-2018* 1–11 (2018).
3. Larson, M., Hoan, L. X. & Hanson, H. Direct Formula to Compute Wave Height and Angle at Incipient Breaking. *J. Waterw. Port Coast. Ocean Eng.* **136**, 119–122 (2010).
4. Medellín, G., Medina, R., Falqués, A. & González, M. Coastline sand waves on a low-energy beach at “El Puntal” spit, Spain. *Mar. Geol.* **250**, 143–156 (2008).
5. Falqués, A. & Calvete, D. Large-scale dynamics of sandy coastlines: Diffusivity and instability. *J. Geophys. Res. Oceans* **110**, (2005).
6. Warrick, J. A. *et al.* Beach morphology and change along the mixed grain-size delta of the dammed Elwha River, Washington. *Geomorphology* **111**, 136–148 (2009).

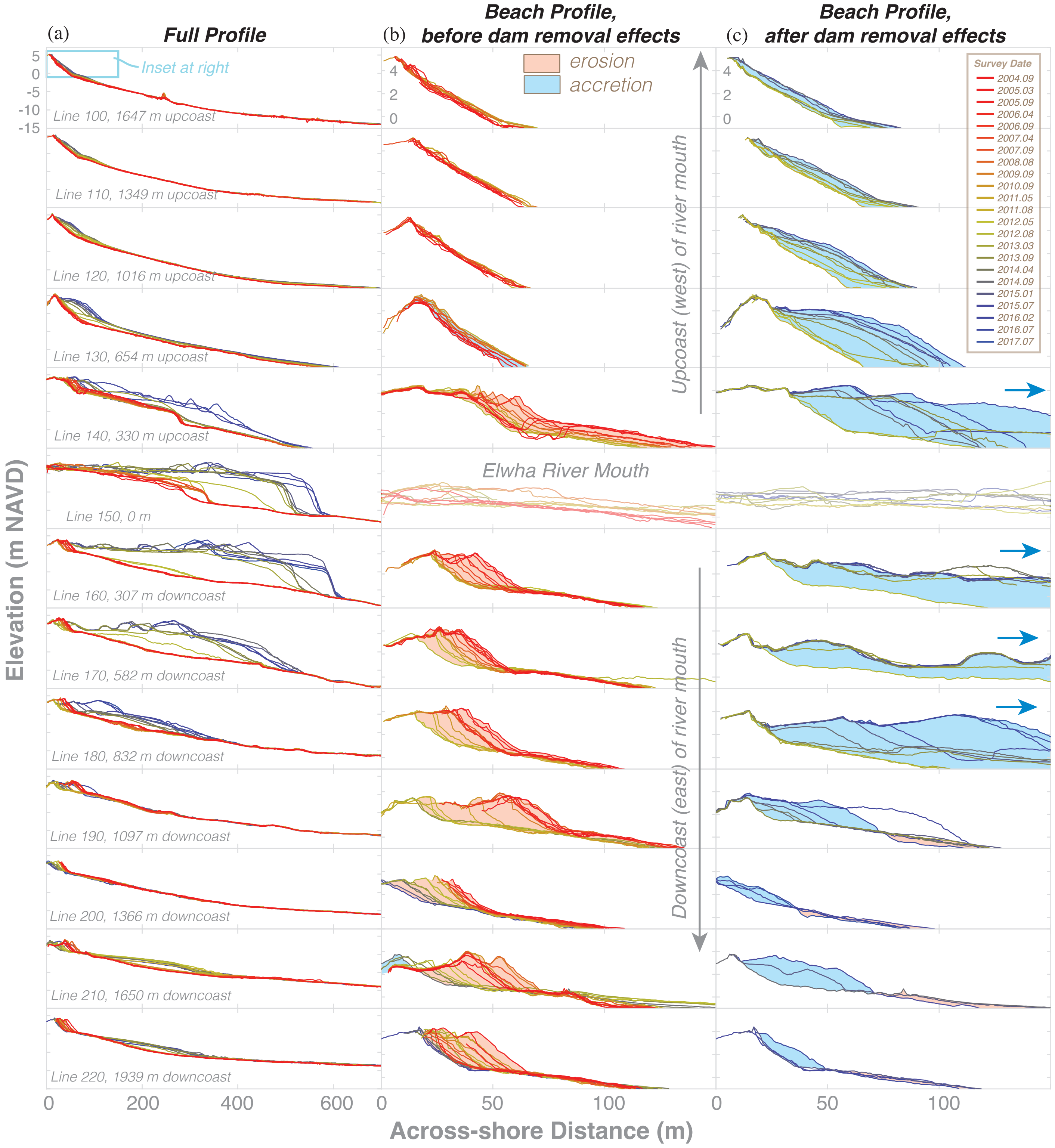

Supplemental Figure 2. Example topographic-bathymetric profiles of the Elwha River delta shoreline. (a) Full profiles highlighting both subaerial and submarine changes with time. (b-c) Inset profiles (see top panel of (a) for inset area) highlighting the shoreface changes both (b) before and (c) after the dam removal effects. Areas of erosion and accretion are highlighted in (b-c) with shading.

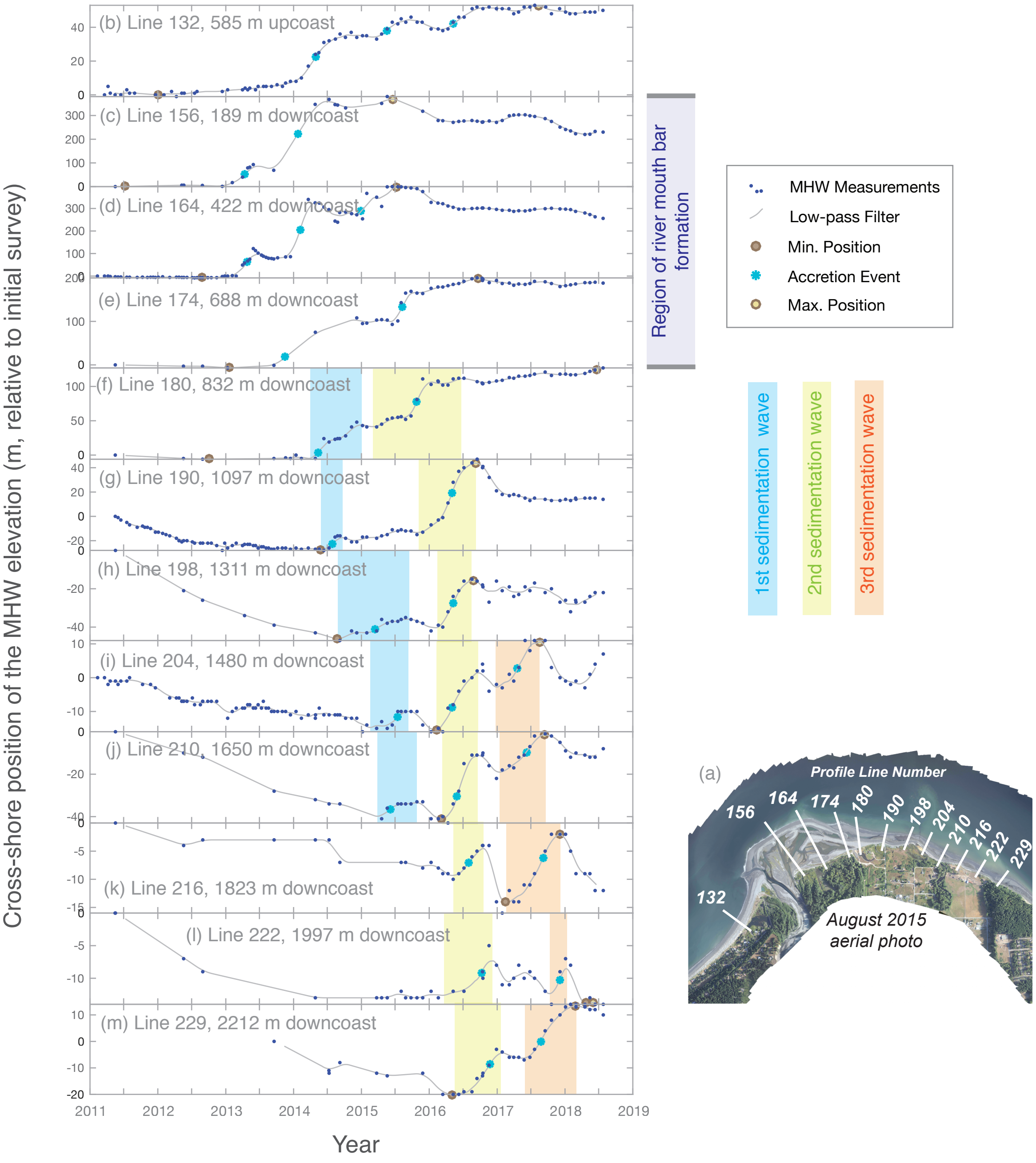

Figure S3. Time series of shoreline positions dervied from regularly surveyed topographic profiles along the Elwha River delta. (a) Location of the twelve profiles. (b-m) Small symbols show the across-shore postions of mean high water (MHW) elevations from topographic profiles; lines provide quarterly moving averages. Accretionary events and across-shore minimum and maximum are shown with symbols (see Methods). Note that each vertical scale in (b) to (m) are unique. Inset map created with ArcMap version 10.5 (http://desktop.arcgis.com/en/arcmap/) using structure-from-motion products created with Agisoft PhotoScan 1.1.6 through 1.2.6 (http://www.agisoft.com).

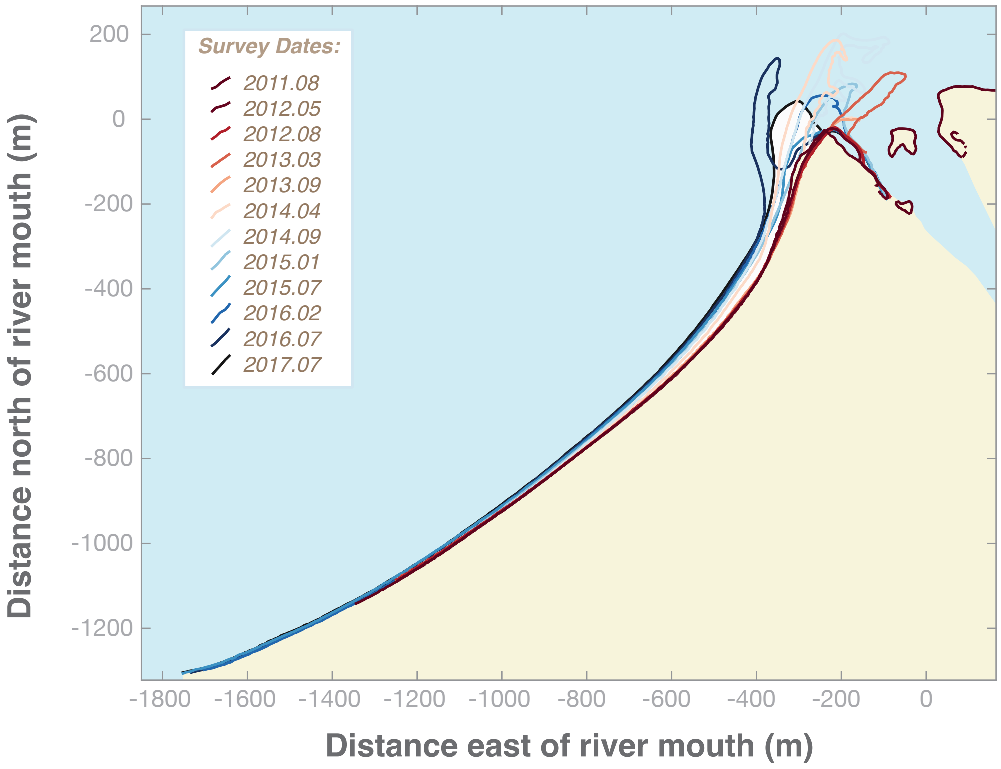

Supplemental Figure S4. Shorelines of the upcoast Elwha River delta before, during and after dam removal showing river bar welding and subsequent sediment waves. Shorelines derived from the MHW positions in the DEMs generated by the topo-bathy surveys.

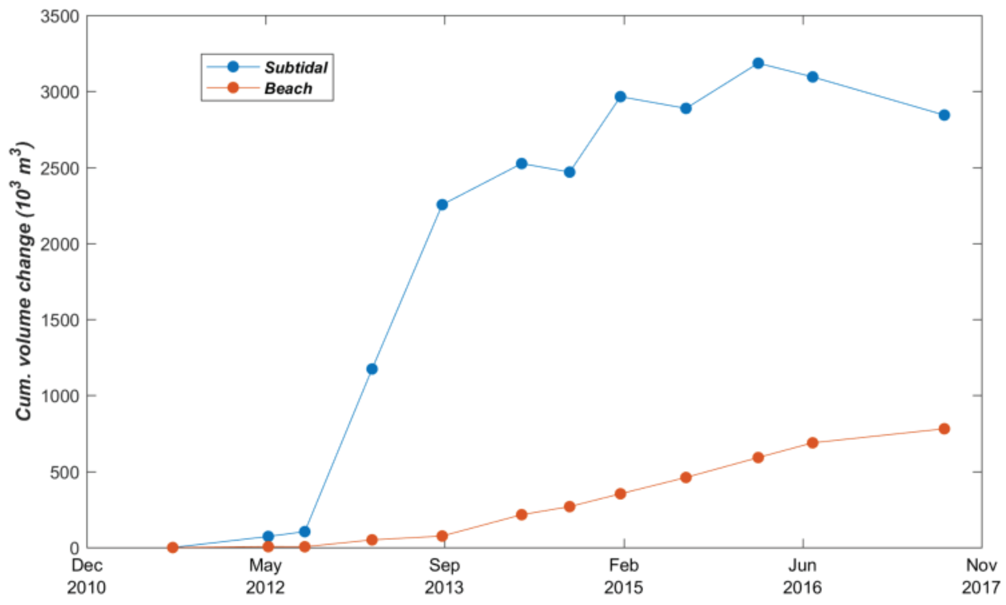

Supplemental Figure S5. Cumulative volume change of the Elwha River delta since the beginning of dam removal (after Gelfenbaum et al., 2015). The subtidal and beach portions of the delta are separated at the mean lower low water (MLLW) tidal datum.

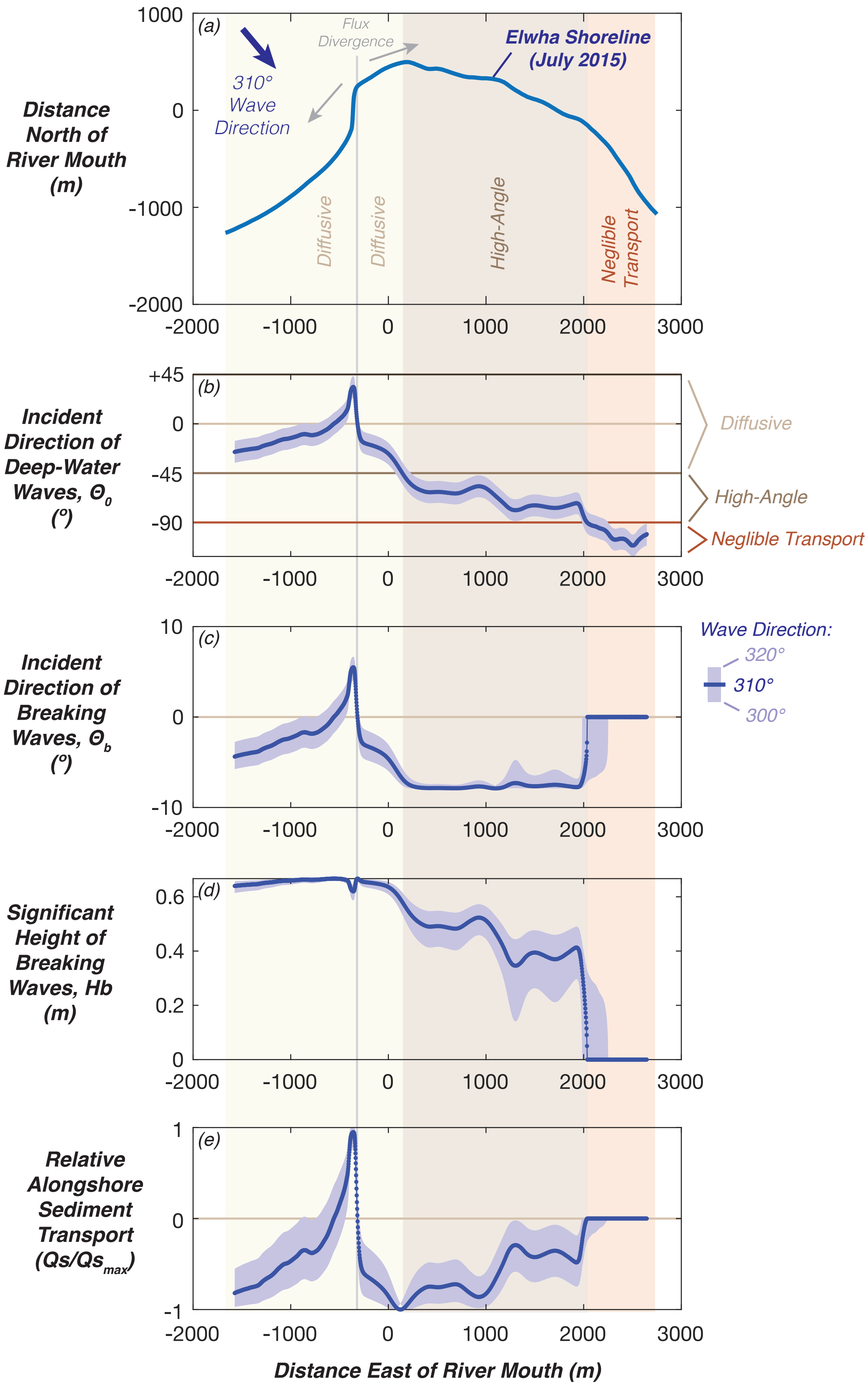

Suppleental Figure S6. Modeled coastal wave characteristics and littoral sediment transport for the shoreline of the Elwha River delta using the formulations of Ashton and Murray (2006) and Larson et al. (2010)and the July 2015 shoreline as described in the text. Shaded regions show different littoral sediment tranport styles using the theory of Ashton and Murray (2006), which are used to compare and contrast with observations presented in our paper.

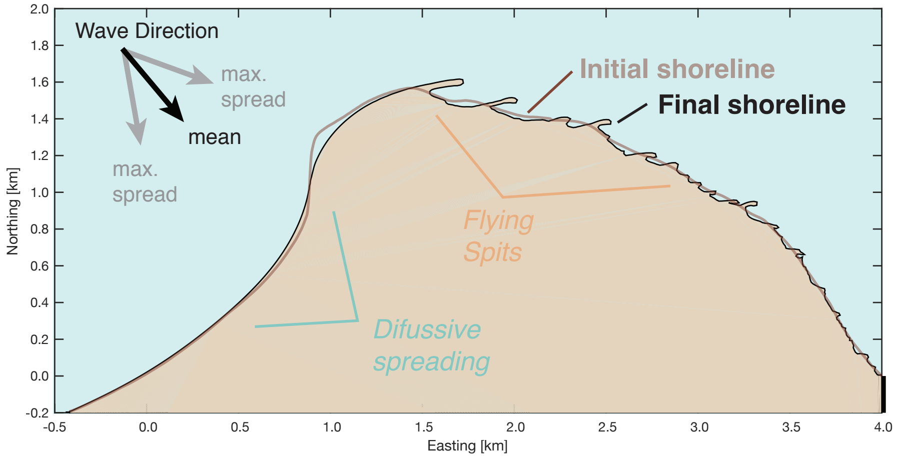

Supplemental Figure S7. Modeled shoreline evolution for the Elwha River delta using the formulations of Ashton and Murray (2006) within the coastal evolution model of Roelvink et al. (2018). Model was run for a 6-month interval using constant 0.5 m waves (see text).
